# A novel complement-fixing IgM antibody targeting GPC1 as a useful immunotherapeutic strategy for the treatment of pancreatic ductal adenocarcinoma

**DOI:** 10.1186/s12967-023-04745-9

**Published:** 2023-11-28

**Authors:** Davide Busato, Sara Capolla, Paolo Durigutto, Monica Mossenta, Sara Bozzer, Daniele Sblattero, Paolo Macor, Michele Dal Bo, Giuseppe Toffoli

**Affiliations:** 1 Experimental and Clinical Pharmacology, Centro Di Riferimento Oncologico (CRO) Di Aviano IRCCS, 33081 Aviano, Italy; 2 Department of Life Sciences, University of Trieste, 34127 Trieste, Italy

**Keywords:** PDAC, GPC1, IgM, Complement System, Immunotherapy

## Abstract

**Background:**

Pancreatic ductal adenocarcinoma (PDAC) is one of the most aggressive cancers with a very low survival rate at 5 years. The use of chemotherapeutic agents results in only modest prolongation of survival and is generally associated with the occurrence of toxicity effects. Antibody-based immunotherapy has been proposed for the treatment of PDAC, but its efficacy has so far proved limited. The proteoglycan glypican-1 (GPC1) may be a useful immunotherapeutic target because it is highly expressed on the surface of PDAC cells, whereas it is not expressed or is expressed at very low levels in benign neoplastic lesions, chronic pancreatitis, and normal adult tissues. Here, we developed and characterized a specific mouse IgM antibody (AT101) targeting GPC1.

**Methods:**

We developed a mouse monoclonal antibody of the IgM class directed against an epitope of GPC1 in close proximity to the cell membrane. For this purpose, a 46 amino acid long peptide of the C-terminal region was used to immunize mice by an *in-vivo* electroporation protocol followed by serum titer and hybridoma formation.

**Results:**

The ability of AT101 to bind the GPC1 protein was demonstrated by ELISA, and by flow cytometry and immunofluorescence analysis in the GPC1-expressing "PDAC-like" BXPC3 cell line. *In-vivo* experiments in the BXPC3 xenograft model showed that AT101 was able to bind GPC1 on the cell surface and accumulate in the BXPC3 tumor masses. *Ex-vivo* analyses of BXPC3 tumor masses showed that AT101 was able to recruit immunological effectors (complement system components, NK cells, macrophages) to the tumor site and damage PDAC tumor tissue. *In-vivo* treatment with AT101 reduced tumor growth and prolonged survival of mice with BXPC3 tumor (p < 0.0001).

**Conclusions:**

These results indicate that AT101, an IgM specific for an epitope of GPC1 close to PDAC cell surface, is a promising immunotherapeutic agent for GPC1-expressing PDAC, being able to selectively activate the complement system and recruit effector cells in the tumor microenvironment, thus allowing to reduce tumor mass growth and improve survival in treated mice.

**Supplementary Information:**

The online version contains supplementary material available at 10.1186/s12967-023-04745-9.

## Introduction

Pancreatic ductal adenocarcinoma (PDAC) is one of the deadliest cancers with a survival rate of less than 10% at 5 years and represents a major unmet medical need [1]. Unfortunately, the majority of PDAC patients are diagnosed when surgery is not possible and the only therapeutic option remains chemotherapy [2]. Gemcitabine is the most commonly used agent; its administration has also been suggested in combination with albumin-linked paclitaxel [3]. FOLFIRINOX (5-Fluorouracil, Leucovorin, Irinotecan, and Oxaliplatin), as an alternative chemotherapeutic combination strategy, has been shown to be effective in metastatic disease [3]. Nevertheless, the use of chemotherapeutic agents has generally shown only a modest improvement in survival, but is often associated with severe toxicity events [3].

The ability to distinguish cancer cells from healthy tissues is the main goal of immunotherapeutics, which selectively kill tumor cells and reduce toxicity events through targeted activation of the immune system [2, 3]. To this end, monoclonal antibodies (mAbs) have been approved for several types of solid cancers. Antibody-based immunotherapy for the treatment of PDAC, targeting different tumor-associated antigens (TAA), has been proposed, but its efficacy has thus far proven limited [4]. Problems to overcome with this therapeutic approach are the insufficient activation of the immune system but also the immunosuppressive state of the PDAC microenvironment as well as its high content of desmoplastic tissue, leading to impaired drug delivery [4, 5].

One of the mechanisms of action exploited by therapeutic mAbs is complement-dependent cytotoxicity (CDC), which involves activation of the classical pathway of the complement system (CS). The CS can kill cancer cells directly, but can also recruit effector cells of the immune system that contribute to the killing of cancer cells via antibody-dependent cytotoxicity (ADCC) or phagocytosis [6, 7]. The CS has a clear advantage over cytotoxic cells as a defense system, as it consists of soluble molecules that can easily reach and diffuse into the tumor mass; it may be particularly important in the context of PDAC desmoplastic tissue. In addition, the components of CS are readily available as a first line of defense as they are locally synthesized by many cell types, such as macrophages, fibroblasts, and endothelial cells. Several neoplastic cells have also been shown to synthesize and secrete components of CS [8]. Direct killing of tumor cells by the membrane attack complex is one of the mechanisms used by CS to control tumor growth. However, CS can also exert its antitumor activity through additional non-cytotoxic effects. For example, C3b deposited on tumor cells promotes the binding and activation of effector immune cells, including phagocytes and natural killer (NK) cells expressing complement receptor 3 (CR3 -CD11b-CD18), resulting in complement-dependent cell cytotoxicity (CDCC) [7].

Although the IgG isotype represents the majority of mAbs approved for cancer immunotherapy and their activity is usually also associated with activation of the CS, the IgM isotype may be a better alternative due to its higher avidity for the target and because it is the most efficient CS activator [9, 10]. Indeed, the multimeric IgM exploits the proximity of multiple Fc that can efficiently bind and activate C1, the first component of the classical pathway of the CS cascade [6, 7, 9], which eventually induces CDC, recruits inflammatory cells such as macrophages and NK cells and also causes CDCC [7, 11, 12]. In this context, it is of interest that several mAbs of the IgM isotype have been investigated in recent phase I clinical trials and showed promising antitumor activity [13–17].

Among the various TAA, one of the possible candidates is glypican-1 (GPC1), which is highly expressed in PDAC tumor tissues and is not expressed or is expressed at very low levels in normal pancreatic tissue and in chronic pancreatitis [18–20]. GPC1 is expressed in the embryo, where it is essential for development, but its expression is very limited in most adult tissues [21, 22]. GPC1 is a cell surface proteoglycan composed of the first 23 amino acids representing the secretory signaling peptide, the N-terminal region localized between amino acids 24 and 474, and the C-terminal region localized between amino acids 475 and 530, and has a total molecular weight of 62 kDa [23]. The GPI anchor linked with the C-terminal region is essential for the binding to the cell membrane [23].

As for the functional aspect, GPC1 could represent an interesting TAA to target with immunotherapeutics, also because it is associated with several growth factors such as fibroblast growth factor 2 (FGF2), vascular endothelial growth factor (VEGF), heparin-binding EGF-like growth factor (HB-EGF) and transforming growth factor-β (TGF-β), which are involved in cancer cell proliferation, angiogenesis and metastasis [24]. Considering the potential impact on interactions with the tumor microenvironment, the expression of GPC1 has also been observed in cancer-associated fibroblasts (CAF) involved in stroma formation, which is associated with an immunosuppressive state that supports cancer progression [24].

The high expression of GPC1 on the surface of PDAC cells, its involvement in tumor progression and its role in the immunosuppressive tumor microenvironment prompted the development of a novel anti-GPC1 IgM mAb (AT101) for the treatment of PDAC patients. Unlike other antibodies against the same target, we focused on an epitope as close to the cell membrane as possible to maximize the activation of CS on tumor cells. Indeed, it has already been shown that several factors may play an important role in promoting more efficient CS by therapeutic mAbs, including the proximity of the target epitopes to the cell surface [25]. In the present study, we demonstrated that AT101 can be readily produced and purified; it specifically recognizes PDAC cells, both *in-vitro* and in a subcutaneous mouse PDAC xenograft model in athymic nude mice. AT101 leads to potent local activation of CS on the cell surface, causing lysis of cancer cells and recruitment of NK cells and macrophages to the tumor microenvironment, ultimately leading to a significant reduction in PDAC tumor growth and increasing survival of all treated mice.

## Methods

### Cell culture

The culture media employed for culturing the cell lines were: DMEM high glucose with L-glutamine and sodium pyruvate (DMEM) (Euroclone S.p.A., Italy); RPMI 1640 with L-glutamine (RPMI) (Euroclone S.p.A., Italy); Hybridoma serum free medium (Hybridoma SFM) with L-glutamine (Gibco, Invitrogen, Italy). DMEM and RPMI were supplemented with 10% of fetal bovine serum (FBS) (Microgem laboratory research, Italy), 1% of MEM non-essential aminoacids (Euroclone S.p.A., Italy), and 1% of penicillin–streptomycin solution (Euroclone S.p.A., Italy). Hybridoma SFM was supplemented with HT (Gibco, Invitrogen, Italy) at a final concentration of 1%, and with 1% of penicillin–streptomycin solution (Euroclone S.p.A., Italy).

BXPC3 cell line (human pancreatic ductal adenocarcinoma) (RRID: CVCL_0186) was purchased from ATCC and cultured in DMEM. Jurkat cell line (human acute T cell leukemia) (RRID: CVCL_0065) was purchased from DSMZ and cultured in RPMI. Hybridoma cells were cultured in hybridoma SFM.

All the cell lines were maintained at 37 °C in a humidified incubator (SANYO, Japan) with 95% air and 5% CO_2_.

### Immunoglobulin variable heavy and light chain sequencing

RNA extraction and retrotranscription into cDNA were performed using TRIsure™ (Bioline, TN, US) and SuperScript® III First-Strand Synthesis System for RT-PCR (ThermoFisher Scientific, Waltham, MA, US), respectively. The sequence of the primers used for the light variable (VL) chain and the heavy variable (VH) chain amplification are reported in Additional file 1: Table S1 and Additional file 1: Table S2 respectively [26, 27]. For this purpose, PCRBIO HIFI^™^ polymerase (Resnova, Italy) was used. The PCR products obtained were then purified using the PCR Clean-up kit (Sigma-Aldrich, Italy), and the corresponding sequences were obtained using the Mix2Seq kit (Eurofins, Italy). All procedures were performed according to the procedures described by the manufacturers. VH and VL sequencing analysis was performed using the international ImmunoGeneTics information system^®^ (IMGT^®^).

### Immunofluorescence analysis

For immunofluorescence analysis (IF) of BXPC3 and Jurkat cells, primary antibodies employed were anti-GPC1 commercial (Thermo Fisher Scientific, Italy, Cat. No. PA5-28,055) diluted 1:100 and AT101 50 µg/ml. Secondary antibodies employed were: anti-mouse IgM 594 conjugated (Bethyl, Fortis Life Science, MA, USA, Cat. No. A90-201D4) diluted 1:250; anti-rabbit IgG 594 conjugated (Bethyl, Fortis Life Science, MA, USA, Cat. No. A120-111D4) diluted 1:100.

For IF in organs and BXPC3 tumors, primary antibodies employed were: anti-GPC1 (Thermo Fisher Scientific, Italy, Cat. No. PA5-28,055); AT101 25 µg/ml; anti-Von Willebrand Factor (VWF) (Agilent Dako, CA, USA Cat.No. A008202-2) diluted 1:400; anti-C1q (HycultBiotech, The Netherlands, Cat. No. HP8021) diluted 1:50; anti-C3 (HycultBiotech, The Netherlands, Cat. No. HP8022) diluted 1:50; anti-C9 (kindly provided by Prof. Daha) diluted 1:25; anti-CD14 (Santa Cruz Biotechnology, TX, USA, Cat. No. sc-58951) diluted 1:40; anti-CD56 (Advanced BioDesign, France, Cat. No. 748094) diluted 1:100; anti-IgM (Meloy Springfield, VA, USA, Cat. No. B107) diluted 1:400. Secondary antibodies employed were: anti-mouse IgM 488 conjugated (Bethyl, Fortis Life Science, MA, USA, Cat. No. A90-201D2) diluted 1:250; anti-rabbit IgG 488 conjugated (Bethyl, Fortis Life Science, MA, USA, Cat. No. A120-212D2) diluted 1:100; anti-goat IgG 488 conjugated (Invitrogen, Thermo Fisher, Scientific, Italy, Cat. No A32814) diluted 1:300; anti-rat IgG FITC conjugated (Merck, Germany, Cat. No. F6258) diluted 1:100.

For quantitative analysis, images were also analyzed using Image-J software. For the analysis, at least 15 images, from 3 different slides for each condition, were performed; two different Region of Interest (ROIs) were set on a picture: the first one on nuclei fluorescence and the other on the fluorescence derived from analyzed target. Data are expressed as normalized fluorescence (protein/nuclei).

### Establishment of PDAC xenograft murine model

The *in-vivo* studies were conducted under the authorization of the Italian Ministry of Health No. 788/2015- PR. All procedures were performed on female Nude-Foxn1nu mice at 8 weeks of age provided by Inotiv (order number: 069). For induction of PDAC tumor mass, 4 million BXPC3 cells at a concentration of 2 million/50 µl in PBS were injected subcutaneously into the flank of each mouse. The mice were monitored three times a week for tumor mass development and assessment of general wellness.

### In-vivo and ex-vivo biodistribution studies of AT101

Biodistribution studies were performed comparing AT101 with unspecific murine IgM; the two experimental groups consisted of 4 animals each. Mice with a BXPC3 tumor mass with a volume of 196 mm^3^ were injected with the molecule of interest into the tail vein. Tumor volumes were measured with a caliper instrument and dimensions were calculated using the following formula: (length × width^2^)/2. For visualization with the VIVOVISION IVIS^®^Lumina (IVIS) *in-vivo* imaging system, each preparation was conjugated with Cy5.5 and injected at a concentration of 1 nmol Cy5.5 (see Additional materials and methods). For the assessment of biodistribution by IVIS, mice were anesthetized with a solution of nimatek (Dechra) and medetor (Virbac) and reawakened with antisedan (Orion). Biodistribution was assessed every 24 h to 96 h considering the average efficiency in the region of interest (ROI). After 96 h, mice were sacrificed by cervical dislocation. After the sacrifice, tumor, pancreas, spleen, ovary, intestine, kidneys, liver, heart, lungs, and brain were collected and analyzed *ex-vivo* by IVIS considering average efficiency and by IF.

### In-vivo evaluation of the efficacy of AT101 as an immunotherapeutic agent

In this study, 2 groups of 7 mice with a subcutaneous model of PDAC received AT101 or PBS (control group). The study started as soon as the mice developed a subcutaneous BXPC3 tumor mass with a volume of 75 mm^3^. Tumor volumes were measured with a caliper and dimensions were calculated using the following formula (length × width^2^)/2. AT101 was administered intraperitoneally (i.p.) twice weekly at a dose of 1.5 mg/kg. Tumor size and general wellness (body score condition (BCS), diarrhea, vomiting, cramps, dehydration, tachypnoea, dyspnea, motionless) were assessed three times weekly. AT101 was administered until day 42 and the mice were sacrificed on day 50, the endpoint of the experiment. The humanitarian endpoints that led to the euthanasia of the mice were: a tumor size greater than 12 mm or ulceration of the tumor masses.

Further details regarding the materials and methods used are provided as Additional materials and methods.

## Results

### The AT101 IgM recognizes the GPC1 protein in-vitro

**Fig. 1:**
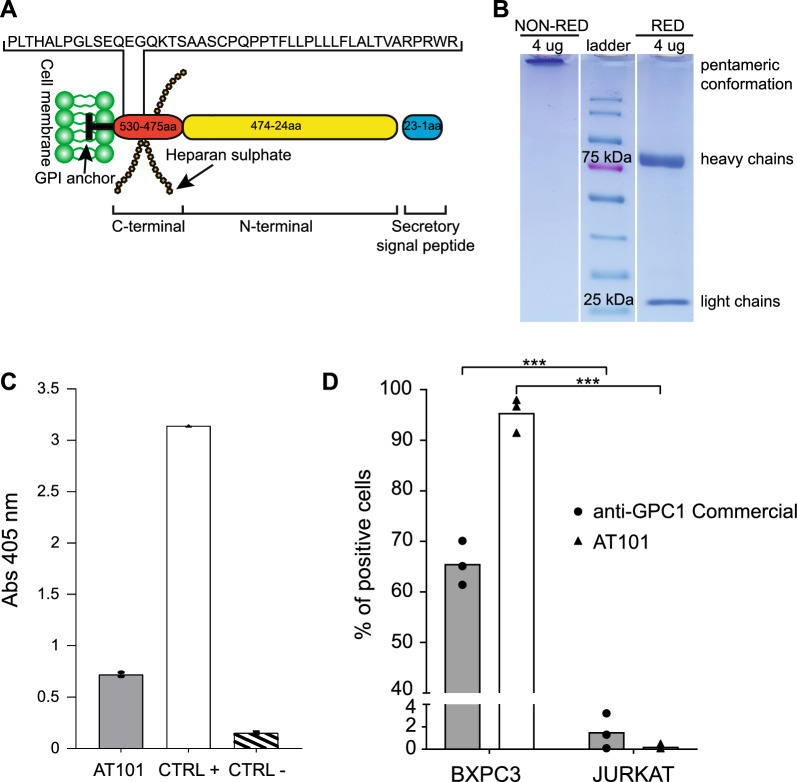
*In-vitro* characterization of AT101. **A** Schematic representation of GPC1 protein and visualization of the aminoacids employed for the mouse immunization. **B** SDS-PAGE analysis performed in non-reducing and reducing condition to determine the IgM nature of AT101. **C** Elisa to assess the GPC1 binding activity of AT101, the positive control (CTRL +) was represented by serum of mice immunized with GPC1, the negative control (CTRL-) was performed by only using the goat anti-mouse IgG/IgM antibody conjugated with alkaline phosphatase (AP) without AT101 (n = 3).** D** Flow cytometry analysis to evaluate GPC1 expression in BXPC3 and Jurkat cells using AT101 and the commercial anti-GPC1 antibody as positive control (n = 3). ***, 0.001 < p ≤ 0.0001

The peculiar properties of GPC1 as a specific PDAC TAA [21, 24, 28], prompted us to develop a mouse mAb of the IgM class directed against an epitope of GPC1 in close proximity to the cell membrane in order to propose an antibody-based immunotherapeutic. In collaboration with Takis Biotech S.r.l. (Rome, Italy), a 46 amino acid long peptide of the C-terminal region was used to immunize mice (Fig. 1A), using an *in-vivo* electroporation protocol followed by serum titer and hybridoma formation. We chose to use the C-terminal region because this part of the GPC1 sequence is more specific than the N-terminal region, which is the most homologous region among the glypican family components. Furthermore, unlike the C-terminal region, the N-terminal region can be cleaved, thus becoming not available for the binding of antibodies directed against the surface of the GPC1 protein [29, 30]. In this way we obtained AT101, an antibody of the murine IgM class specifically directed against the GPC1 protein. The sequence of its variable regions was first obtained by using degenerate primers specific for the mouse VH and VL chains; the amino acid sequences are listed in Additional file 1: Table S3 (patent application number 102022000021546 Italian Ministry of Economic Development). Hybridoma cells were conditioned to grow in serum-free medium and anti-GPC1 IgM was purified from the culture medium by affinity chromatography. SDS-PAGE was used to visualize the purified form of the AT101 antibody. Specifically, the IgM nature of AT101 was visualized in Fig. 1B, in which the running pattern under non-reducing conditions showed a single band attributable to the pentameric IgM conformation; the use of reducing conditions showed two bands attributable to the heavy chains and light chains of AT101. No degradation products or unwanted proteins were documented.

Binding of AT101 to the target protein was first detected *in-vitro* by ELISA on wells coated with GPC1 and compared with a serum from mice immunized with GPC1. The results confirmed that AT101 binds the GPC1 protein (Fig. 1C). To investigate the ability of AT101 to bind GPC1 also on the cell surface, AT101 was tested by flow cytometry using the GPC1-expressing BXPC3 cell line or the GPC1-negative Jurkat cell line. Positivity and negativity for GPC1 expression of the cells were controlled with the commercial anti-GPC1 polyclonal antibody (see methods). As shown in Fig. 1D, AT101 was able to recognize the GPC1 protein in the GPC1-expressing BXPC3 cells by flow cytometry. On the other hand, no signals corresponding to the presence of the GPC1 protein were obtained when flow cytometry experiments were performed with the cells of the Jurkat cell line.

The effectiveness of AT101 in recognizing the GPC1 protein on the cell surface was also investigated by IF. First, the localization of GPC1 was visualized with the commercial antibody (Additional file 1: Figure S1), as already described [24, 31]. When the AT101 was employed, a distinct cell membrane localization on BXPC3 cells was shown. As expected, GPC1 expression was not detected on Jurkat cells with either the anti-GPC1 commercial antibody or the AT101 antibody (Additional file 1: Figure S1).

Altogether these results demonstrated that AT101 can be easily produced and purified and that it is able to specifically target the surface of GPC1 expressing cells.

### The PDAC xenograft mouse model obtained by BXPC3 injection expresses GPC1 and exhibits vascularization

**Fig. 2:**
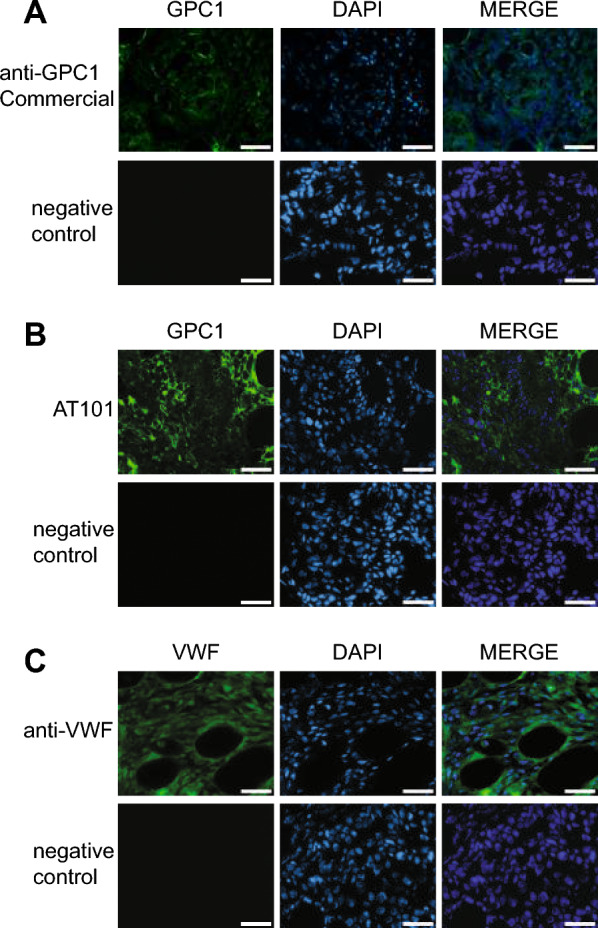
*Ex-vivo* characterization of the PDAC xenograft murine model. **A, B** IF to evaluate GPC1 expression using AT101 and the commercial anti-GPC1 antibody as positive control, respectively. In green the signal related to GPC1 protein and in blue the nuclei. Scale bar: 25 µm. **C** IF to evaluate VWF expression in order to determine the presence of vascularization. In green the signal related to VWF protein and in blue the nuclei. Scale bar: 25 µm

To investigate the properties of AT101 when injected *in-vivo*, a xenograft mouse model of PDAC was created by subcutaneously injecting BXPC3 cells into the flank of athymic mice and then analyzed for the expression of GPC1. The mice were sacrificed when the tumor mass reached a volume of 500 mm^3^. The tumor masses were then removed and analyzed using IF. As shown in Fig. 2A, the BXPC3 cells of the explanted tumor masses retained GPC1 expression as determined with the commercial anti-GPC1 polyclonal antibody. Furthermore, the GPC1 expression pattern obtained with AT101 was similar to that obtained with the commercial anti-GPC1 antibody (Fig. 2B). These results also indicated that AT101 is able to recognize GPC1 even when used on frozen tissue.

Finally, the presence of vascularization, which is required for IgM accumulation in BXPC3 tumor tissue, was investigated by evaluating the expression of VWF, a typical marker for endothelial cells. As shown in Fig. 2C, the formation of vessels in the context of BXPC3 tumor masses was defined by the VWF expression pattern.

Overall, the results of the IF analysis of BXPC3 tumor masses suggested that the established PDAC xenograft model was useful to study the biodistribution, specificity and efficacy of AT101 *in-vivo*.

### AT101 recognizes GPC1 protein in-vivo

**Fig. 3:**
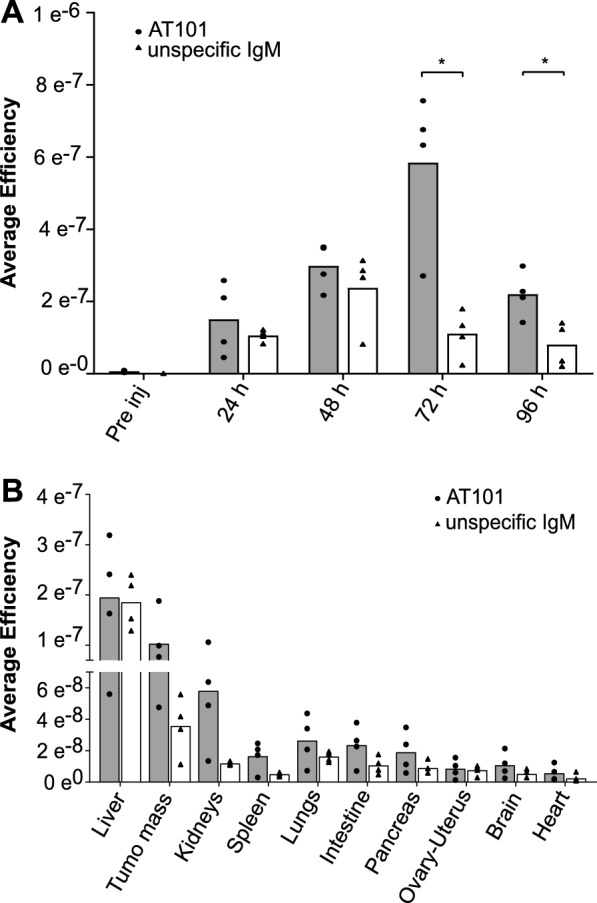
*In-vivo* and *ex-vivo* biodistribution of AT101. **A** bar chart of the *in-vivo* biodistribution, using VIVOVISION IVIS^®^Lumina, of AT101 (1 nmol of Cy5.5) in comparison with the negative control unspecific IgM (1 nmol of Cy5.5). Data are reported as average efficiency mean ± SD (n = 4). P-value was calculated using t-test. ns: ≥ 0.05; *: 0.05 < p ≤ 0.01; **: 0.01 < p ≤ 0.001. **B** bar chart of the *ex-vivo* biodistribution, using VIVOVISION IVIS®Lumina, of AT101 (1 nmol of Cy5.5) in comparison with the negative control unspecific IgM (1 nmol of Cy5.5). Data are reported as average efficiency mean ± SD (n = 4). P-value was calculated using t-test. ns: ≥ 0.05

To evaluate the ability of AT101 to target the tumor mass after injection into the bloodstream and its overall biodistribution, an *in-vivo* time course experiment with signal detection from 0 to 96 h and signal evaluation every 24 h was performed using the PDAC xenograft mouse model. AT101 was compared with unspecific mouse IgM. Both molecules were conjugated to Cy5.5. A single dose (1.5 mg/kg) was injected i.v. into two different groups of 4 BXPC3 tumor-bearing mice, and biodistribution was followed for 96 h. AT101 showed accumulation at the tumor site starting 24 h after injection and lasting until 96 h, with a fluorescence peak at 72 h. Overall, the accumulation pattern of AT101 was significantly different from that of labelled non-specific IgM (p = 0.017 at 72 h and 0.019 at 96 h, Fig. 3A).

In general, *ex-vivo* analysis of organs and tumor masses explanted after 96 h confirmed the presence of the labelled antibodies in the tumor masses and in the liver, suggesting hepatic clearance of the injected IgM (Fig. 3B).

### AT101 induces an immune response against BXPC3 tumor masses

**Fig. 4:**
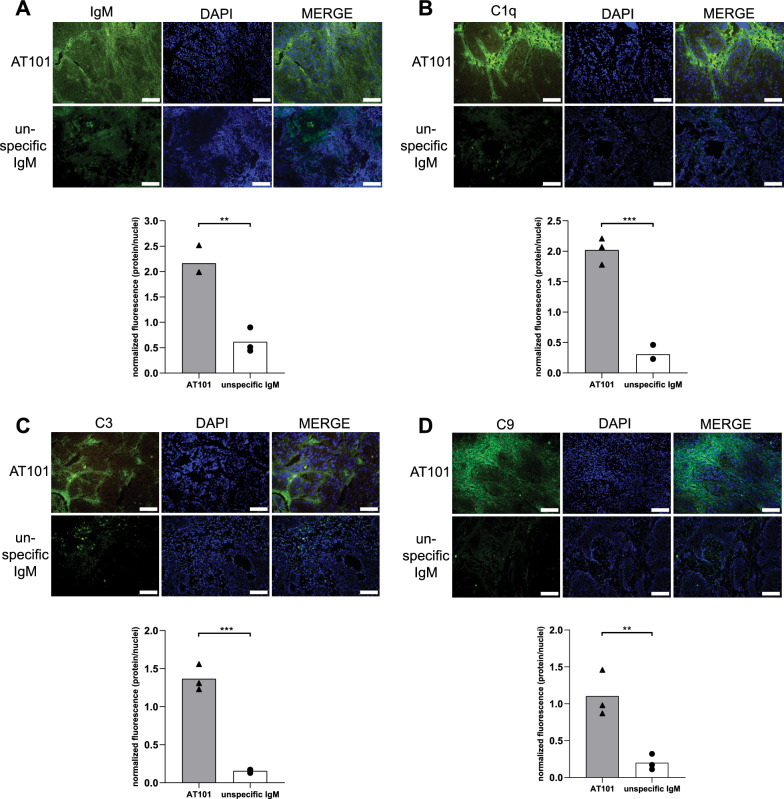
*Ex-vivo* evaluation of the complement system activation at tumor site. **A** Upper panel: IF to evaluate the accumulation of IgM in the two experimental groups (AT101 treated mice and unspecific IgM treated mice). In green the signal related to the IgM and in blue the nuclei. Scale bar: 100 µm. Lower panel: bar chart representing the quantification of fluorescence, data are expressed as normalized fluorescence (protein/nuclei). **B** Upper panel: IF to evaluate C1q complement protein in the two experimental groups (AT101 treated mice and unspecific IgM treated mice). In green the signal related to C1q protein and in blue the nuclei. Scale bar: 100 µm. Lower panel: bar chart representing the quantification of fluorescence, data are expressed as normalized fluorescence (protein/nuclei). **C** Upper panel: IF to evaluate C3 complement protein in the two experimental groups (AT101 treated mice and unspecific IgM treated mice). In green the signal related to C3 protein and in blue the nuclei. Scale bar: 100 µm. Lower panel: bar chart representing the quantification of fluorescence, data are expressed as normalized fluorescence (protein/nuclei). **D** Upper panel: IF to evaluate C9 complement protein in the two experimental groups (AT101 treated mice and unspecific IgM treated mice). In green the signal related to C9 protein and in blue the nuclei. Scale bar: 100 µm. Lower panel: bar chart representing the quantification of fluorescence, data are expressed as normalized fluorescence (protein/nuclei)

The higher accumulation of IgM in BXPC3 tumor masses shown *in-vivo* and *ex-vivo* in mice treated with AT101 compared to mice treated with unspecific IgM was also confirmed by IF by evaluating explanted tumor masses after 96 h, both visualizing the figures or analyzing fluorescence signal in the different samples (Fig. 4A).

The presence of IgM says nothing about their binding and activation of the CS. To this end, we examined the deposition of C1q, C3 and C9 in the tumor masses using IF. As shown in Fig. 4B–D evidences of C1q binding as well as C3, C9 deposition was clearly documented in tumor sections from mice treated with AT101, whereas it was not visible in samples obtained from unspecific IgM-treated mice. These data were confirmed also by analyzing fluorescence signals (Fig. 4B).

**Fig. 5:**
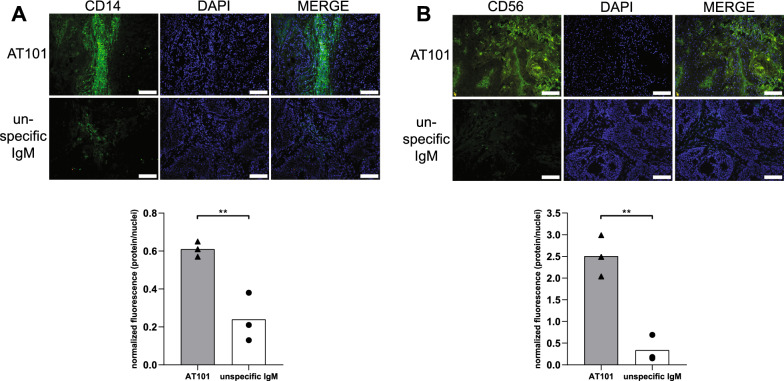
*Ex-vivo* evaluation of the immunological recruitment at tumor site. **A** Upper panel: IF to evaluate CD14 protein in the two experimental groups (AT101 treated mice and unspecific IgM treated mice). In green the signal related to CD14 protein and in blue the nuclei. Scale bar: 100 µm. Lower panel: bar chart representing the quantification of fluorescence, data are expressed as normalized fluorescence (protein/nuclei). **B** Upper panel: IF to evaluate CD56 protein in the two experimental groups (AT101 treated mice and unspecific IgM treated mice). In green the signal related to CD56 protein and in blue the nuclei. Scale bar: 100 µm. Lower panel: bar chart representing the quantification of fluorescence, data are expressed as normalized fluorescence (protein/nuclei)

Activation of CS also leads to the formation of anaphylatoxins, such as C3a and C5a, and the associated recruitment of inflammatory cells [7]. Therefore, we analyzed the migration of CD14 + macrophages and CD56 + NK cells using IF. In tumor sections of mice treated with AT101, massive infiltration of both cells was detected, while signals corresponding to CD14 and CD56 expression were barely detectable in tumor sections of mice treated with the unspecific IgM. These data were confirmed also by analyzing fluorescence signals. (Fig. 5A, B). Altogether considered, these results showed the capacity of AT101 to target cancer cells, activate the classical pathway of the CS and cause the recruitment of inflammatory cells in tumor microenvironment.

As a result, the hematoxylin eosin staining showed a strong purple-blue (hematoxylin) coloration in the mice treated with AT101, putatively associated with a high grade of cellular lysis that was not documented in tumor microenvironment of mice treated with unspecific IgM (Additional file 1: Figure S2).

### AT101 controls tumor growth and improves mouse survival

The ability of AT101, in a single injection, to target the GPC1 protein on the cell surface and activate the immune response at the tumor prompted us to investigate its efficacy as an antibody-based immunotherapeutic in the PDAC xenograft mouse model. To determine the administration schedule, we took advantage from biodistribution studies in which AT101 showed an accumulation peak 72 h after injection and then rapid elimination from the BXPC3 tumor mass. In this context, a single dose of 1.5 mg/kg was able to induce activation of CS and recruitment of inflammatory cells as well as lysis of tumor cells. For this reason, AT101 was administered at a concentration of 1.5 mg/kg twice a week for six weeks during the experimental procedure (day 42) to ensure a constant high concentration reaching the cancer cells.

**Fig. 6:**
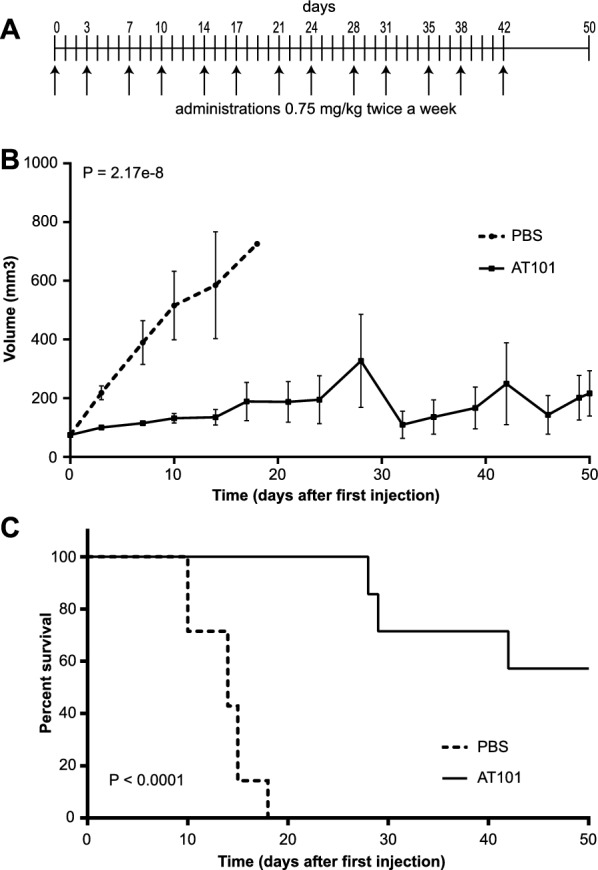
*In-vivo* therapeutic efficacy of AT101. **A** Timeline that recapitulates the scheduling of the treatment. AT101 was administered twice a week at a dosage of 1.5 mg/kg, the last treatment was administered at day 42, the experimental endpoint was set up at day 50. **B** Line graph that recapitulates the tumor growth trend of the two experimental groups. In dashed line the tumor growth trend of the group of mice treated with PBS (n = 7) is reported; in continuous line the tumor growth trend of the group of mice treated with AT101 (n = 7) is reported. Data were represented as mean ± standard error (SE). **C** Kaplan–Meier curve of mouse survival: the survival of mice treated with AT101 was compared with the mice treated with PBS (dashed line). In dashed line the survival curve of the group of mice treated with PBS (n = 7) is reported; in continuous line the survival curve of the group of mice treated with AT101 (n = 7) is reported. P-value < 0.0001 was calculated using log rank test

Two groups of 7 mice were compared. One group was treated intra-peritoneally with AT101, while the second group was treated with PBS as a control group. Treatment started when the tumor reached a volume of 75 mm^3^ and the animals were observed for 50 days from the first treatment (Fig. 6A). Measurement of tumor mass, shown in Fig. 6B, indicates that AT101 reduced tumor growth compared to the control group (p = 2.17 × 10^–8^). The activity of AT101 was clearly noticeable since the first administration. The Kaplan–Meier curves shown in Fig. 6C compared the survival of AT101 treated tumor bearing mice with that of the control group; these data showed that mean survival of AT101 treated mice was significantly longer than that of control mice (median survival of AT101 treated mice not reached versus median survival of PBS treated mice of 14 days; p < 0.0001). Of note, of the 4 AT101-treated mice that reached the experimental endpoint at 50 days, 3 animals showed a reduction in tumor mass, while complete remission was observed in 1 animal. The study showed a promising time window (from day 19 to day 27) in which all PBS treated animals were already euthanized, while treatment with AT101 resulted in 100% of the mice surviving (Fig. 6C). No evidence of toxicity (BCS, diarrhea, vomiting, convulsions, dehydration, tachypnea, dyspnea, motionless) were observed in the group of mice treated with multiple injection of AT101.

## Discussion

In the present study, a novel anti-GPC1 antibody (i.e. AT101) was characterized for the treatment of PDAC. We demonstrated that AT101 is able to: (i) specifically recognize GPC1-expressing cells; ii) specifically accumulate in the tumor microenvironment of a PDAC xenograft mouse model; iii) elicit a very strong immune response; iv) control tumor growth in the xenograft mouse model of PDAC.

PDAC represents one of the most malignant tumor types. In most patients, surgery, the only curative option, is not suitable because the disease is already in an advanced stage at the time of diagnosis [32–35]. At this stage, the therapeutic proposal remains the use of chemotherapy, which unfortunately is not very efficient, due to chemoresistance events, and because it is unable to converge the cytotoxic effect only on the tumor cells, causing frequent and severe side effects [2, 3, 35, 36]. In this scenario, the use of immunotherapeutic agents, such as mAbs, could ensure that antitumor activity is concentrated on tumor cells, reducing the side effects, generally caused by conventional therapy [37, 38]. In the context of PDAC the introduction of mAbs has not provided benefits for the patients. This failure could be ascribable to the immunosuppressive tumor microenvironment and to the desmoplasia condition responsible of an impairment in drug delivery [4, 5] but it could also depend from the target and the type of antibody that was respectively chose and developed. In the present study, GPC1 was defined as a specific PDAC TAA with the following characteristics: (i) localization on the cell surface [23]; (ii) increased expression in PDAC cells compared to low or absent expression in chronic pancreatitis or normal tissues [18–20]; (iii) interaction with various growth factors involved in cell proliferation, angiogenesis and metastasis [18–21, 39, 40]; (iv) the possibility of modulating the tumor microenvironment and reducing the state of desmoplasia, thanks to its expression on CAF [24]. Moreover, the correct epitope on TAA and the mechanism of action activated by mAbs remain important aspects to be considered [7, 8, 25]. In this context, the results of the present study show that targeting GPC1 using an epitope close to the cell membrane with a specific IgM could be a promising therapeutic option for the treatment of PDAC patients.

Here, the well-established hybridoma technique was used for the development of anti-GPC1 [41]. This technique offers the possibility of obtaining a large amount of mAbs with a high degree of specificity and sensitivity, and it is the method by which most FDA-approved mAbs have been obtained [41]. The choice of the region of GPC1 used for immunization of mice was based on several factors, including high sequence homology among members of the glypican family. In this context, it is noteworthy that the C-terminal region is the region with the lowest homology [29, 30]. Another factor is that the close localization of the target region to the cell membrane facilitates the activation of the CS by the antibody [25]. For these reasons, a small portion of the C-terminal region was used for the immunization to increase specificity exclusively for that GPC1 epitope. The decision to develop a murine IgM was also based on the aim to activate the murine CS in the human-mouse model developed to test therapeutic activity.

Hybridoma technology also made it possible to develop a method for the production and purification of AT101 and to guarantee a protein capable of targeting the GPC1 protein; these results were demonstrated by ELISA, flow cytometry and IF, confirming the specificity of AT101 for its target.

In the present study, a subcutaneous mouse model was created in athymic nude mice. Nude mice are characterized by an impairment of the adaptive immune response, but innate immunity (CS, NK cells, neutrophils, and macrophages) remains active. Therefore, the use of nude mice allows the characterization of the immune response to antibody-based immunotherapy, such as the use of AT101 [42, 43]. A subcutaneous model allows easy measurement of the size of tumor masses and facilitates the analysis of accumulation in the tumor microenvironment [44]. In addition, the subcutaneous model generally had a sufficient amount of functional vessels allowing the transport of a molecule, such as IgM, within the tumor mass [44, 45]. Here, the presence of tumor vessels was investigated by determining the expression of VWF, a typical marker for endothelial cells. This result is important because the distribution of IgM is normally restricted to the bloodstream, but increased permeability caused by an inflammatory process or described in tumor vessels allows selective accumulation in these microenvironments [46, 47]. On the contrary, IgG is normally distributed from the blood to all biological fluids [46, 47]. Thus, the use of IgM can also become a mechanism to improve the specificity of the therapeutic approach due to the selective permeability of the tumor microenvironment.

Labelling with a near-infrared fluorescent dye, such as Cy5.5, has been used extensively for biodistribution studies of antibodies and antibody fragments [11, 48, 49]. *In-vivo* characterization of AT101 began with Cy5.5 labelling and injection into tumor-bearing mice to study its biodistribution compared to unspecific murine IgM. AT101 and the unspecific IgM showed a different accumulation profile at the tumor level. Unspecific IgM accumulated in the tumor microenvironment with a pick 48 h after their intravenous administration, demonstrating the possibility that this antibody isotype can migrate through tumor vessels. AT101 showed higher accumulation in the BXPC3 tumor mass compared to unspecific IgM, with a peak at 72 h. These data were also confirmed *ex-vivo* at the end of the study (96 h) and can be linked to the specificity of AT101 for the GPC1 protein expressed in the BXPC3 tumor masses, allowing for durable localization at the tumor site.

Despite the benefits of developing anti-tumor treatments that can take advantage of CS in controlling tumor growth, the involvement of CS mediated function in mAb action is often not yet fully considered. Indeed, it must be taken into account that CS consists of soluble molecules that can easily reach the tumor site and diffuse into the tumor mass [11, 14, 22, 23]. In addition, the components of CS are synthesized locally by various cell types, including macrophages, and they often act as a first line of defense against cancer cells [11]. In this context, however, the presence of IgM not provides information about their binding to cancer cells or about their ability to activate the CS. Only bound IgM (immune complexes) modify their Fc, enable the binding of C1 and activate the classical pathway of the CS [50]. In the present study, C1q, C3 and C9 were detected by IF only in the tumor mass of animals treated with AT101 and not with non-specific IgM, that have no specificity for tumor cells. The presence of C9 indicates full activation of the cascade and formation of the membrane attack complex, which can cause direct death of tumor cells [6]. This result is usually due to lysis of the cell, as documented in this study by hematoxylin eosin staining, where a strong purple-blue staining (hematoxylin) was observed in the AT101-treated mice, which was not documented in the tumor microenvironment of the animals treated with unspecific IgM. The activation of the CS normally alters the microenvironment by producing anaphylatoxins such as C3a and C5a, which are able to activate the endothelial cells of the tumor vessels and recruit leukocytes from the bloodstream [7]. The massive presence of NK cells and macrophages in the tumor sections of mice treated with AT101 is a typical result of this phenomenon [7, 11, 12], which demonstrated the great potential of this anti-GPC1 mAb in coordinating an immune response. This was confirmed by *in-vivo* experiments in which treatment of mice bearing BXPC3 tumors with AT101 was very effective in controlling tumor growth and prolonging survival.

A crucial step for the possibility of clinical application of AT101 is humanization. The ultimate goal of the humanization process is to obtain a humanized mAb with high antigen binding activity and a minimal immunogenicity that prolongs half-life. On the other hand, biophysical properties such as stability and expression yield are important for commercial purposes. From a technical point of view, complementarity-determining regions (CDR)-grafting humanization, in which the whole mouse CDRs are grafted onto an acceptor human framework, is the most commonly used technique [51]. To avoid reducing the potential immunogenicity of framework residues due to somatic mutations containing T cell effector epitopes, the use of frameworks based on human germline sequences or consensus sequences as acceptor human frameworks was introduced [52–54]. Another approach to reduce the potential immunogenicity of non-human CDRs is the use of specificity-determining residues (SDRs), i.e. the minimum CDR residues required for antigen-binding activity, grafted onto the human germline framework [55–57].

Usually, the development and clinical use of therapeutic mAbs mainly focused on humanized or fully human mAbs of the IgG class [9, 10], although several mAbs of the IgM isotype have been investigated in phase I clinical trials [13–17]. Results of the present study suggest that the therapeutic potential of humanized IgM is to be considered for the specific features of this class, i.e. their high avidity and the effective complement activation, that, in other monoclonal antibody models showed therapeutic advantages at least in a pre-clinical phase [9]. On the other hand, whether the creation of an IgG3, capable of recognizing the AT101 epitope would have given a better result than IgM remains to be considered. Moreover, further studies are needed to evaluate if a CAR-T recognizing the AT101 epitope could demonstrate a higher therapeutic potential than a mAb either of the IgM or the IgG class.

## Conclusion

In the present study we showed a novel therapeutic strategy based on the use of AT101, an IgM specific for an epitope of GPC1 close to PDAC cell surface and able to selectively activate the CS and recruit effector cells in the tumor microenvironment, allowing to reduce tumor mass growth and improve survival in all treated mice. AT101 could also provide a platform for other mAb-based approaches, such as antibody–drug conjugates (ADC), drug-loaded nanoparticles or chimeric antigen receptor (CAR) based cell therapies.

### Supplementary Information

**Additional file 1: Table S1.** Primers employed for sequencing of VL gene. **Table S2.** Primers employed for sequencing of VH gene. **Table S3.** Aminoacid sequences of VL and VH chains of AT101. **Figure S1.** Immunofluorescence analysis to evaluate GPC1 expression in BXPC3 and Jurkat using AT101 and the commercial anti-GPC1 antibody as positive control. In red the signal related to GPC1 protein is reported and in blue the signal related to the nuclei is reported. Scale bar: 25 μm. **Figure S2.** Hematoxylin eosin staining to evaluate the possible presence of tissue damage. Purple blue refers to the nuclei, pink refers to the cytoplasms and the extracellular matrix. Scale bar: 100 μm.

## Data Availability

Data are available upon request.

## Abbreviations

PDAC: Pancreatic ductal adenocarcinoma
mAb: Monoclonal antibody
TAA: Tumor associated antigen
CDC: Complement-dependent cytotoxicity
CS: Complement system
ADCC: Antibody-dependent cytotoxicity
CDCC: Complement-dependent cell cytotoxicity
IgM: Immunoglobulin M
NK cells: Natural killer cells
Fc: Fragment crystallizable
GPC1: Glypican-1
FGF2: Fibroblast growth factor 2
VEGF: Vascular endothelial growth factor
HB-EGF: Heparin-binding EGF-like growth factor
TGF-β: Transforming growth factor-β
CAF: Cancer-associated fibroblasts
DMEM: Dulbecco’s modified eagle medium
RPMI1640: Roswell park memorial institute 1640
SFM: Serum free medium
HT: Hypoxanthine and thymidine
ATCC: American type culture collection
DSMZ: Deutsche Sammlung von Mikroorganismen und Zellkulturen
VL: Light variable chain
VH: Heavy variable chain
IgG: Immunoglobulin G
VWF: Von Willebrand factor
FITC: Fluorescein isothiocyanate
ROI: Region of interest
CD14: Cluster of differentiation 14
CD56: Cluster of differentiation 56
PBS: Phosphate-buffered saline
ELISA: Enzyme-linked immunosorbent assay
CDR: Complementarity-determining region
SDR: Specificity-determining residue
ADC: Antibody–drug conjugate
CAR: Chimeric antigen receptor
