# Supplementary material for: A novel complement-fixing IgM antibody targeting GPC1 as a useful immunotherapeutic strategy for the treatment of pancreatic ductal adenocarcinoma
Source: J Transl Med. 2023 Nov 28;21:864. doi: 10.1186/s12967-023-04745-9 (PMC10685509; doi:10.1186/s12967-023-04745-9)
Supplement: Supplementary file 1 — Additional file 1: Table S1. Primers employed for sequencing of VL gene. Table S2. Primers employed for sequencing of VH gene. Table S3. Aminoacid sequences of VL and VH chains of AT101. Figure S1. Immunofluorescence analysis to evaluate GPC1 expression in BXPC3 and Jurkat using AT101 and the commercial anti-GPC1 antibody as positive control. In red the signal related to GPC1 protein is reported and in blue the signal related to the nuclei is reported. Scale bar: 25 μm. Figure S2. Hematoxylin eosin staining to evaluate the possible presence of tissue damage. Purple blue refers to the nuclei, pink refers to the cytoplasms and the extracellular matrix. Scale bar: 100 μm. [file 12967_2023_4745_MOESM1_ESM.pdf]

Additional information

A novel complement-fixing IgM antibody targeting GPC1 for the treatment of pancreatic ductal adenocarcinoma

Description

1 Additional methods

2 Additional tables

3 Additional figure legends

4 Additional figures

1 Additional methods

1.1 mAb purification

The hybridoma supernatant was collected twice weekly, tested for productivity and specificity and finally the AT101 was purified. Purification was performed using Hitrap IgM Purification HP affinity chromatography (Cytiva, United Kingdom). The supernatant was filtered with a 0.45 µm filter before purification. The following buffers were used for purification: binding buffer 20 mM sodium phosphate, 0.8 M $(NH4)_2SO4$, pH 7.5; elution buffer 20 mM sodium phosphate, pH 7.5; regeneration buffer 20 mM sodium phosphate, pH 7.5 with 30% isopropanol. The column was washed with 5 column volumes of each buffer and equilibrated with 5 column volumes of the binding buffer. The sample was added to the column with a syringe. The unbound sample was washed with 15 column volumes of the binding buffer. AT101 was eluted with 12 column volumes of elution buffer. Finally, the column was regenerated with 7 column volumes of regeneration buffer. To replace the elution buffer, dialysis was performed in PBS using a cellulose dialysis membrane with a cut-off of 14000 Da (Merck, Germany).

1.2 SDS PAGE

The purified AT101 was resuspended in reducing or nonreducing sample buffer (Tris 0.35 M pH 6.8, 30% glycerol, 10% SDS, 0.1% bromophenol blue, each with or without 5% 2-mercaptoethanol) and boiled for 5 minutes. Samples were loaded into a sodium dodecyl sulphate – polyacrylamide gel electrophoresis (SDS-PAGE, 12% acrylamide) and run in Biorad vertical electrophoresis chambers 25 (Biorad, Italy) in SDS buffer (2.5 mM Tris; 25 mM glycine pH 8.3 and 0.01% SDS). Proteins were visualized by Coomassie staining: the gel was stained for 2-3 hours with a solution of 0.1% Brilliant blue G250 in 30% methanol and 10% acetic acid, and proteins were visualized after destaining with 30% methanol and 10% acetic acid.

1.3 ELISA

To confirm binding of the anti-GPC1 antibody AT101, wells (Corning-Costar, MA, USA) were first coated with GPC1 (100 ng/well, LSBio, Seattle, WA, USA) overnight (O/N) at 4°C. At the end of incubation, wells were blocked with 2% milk in PBS (Euroclone S.p.A, Italy) for 30 minutes at room temperature (RT). Then, 500 ng of AT101 were added to the wells and incubated for 1 hour at RT. Serum from mice immunized with GPC1 (1:100, Takis S.r.l., Italy) was used as positive control. The negative control (CTRL-) was performed by only using the goat anti-mouse IgG/IgM antibody conjugated with alkaline phosphatase (AP) without AT101. To remove the excess of unbound antibody, three washes with PBS-TWEEN20 0.1% and three washes with PBS were performed. To detect binding of AT101 to GPC1, a goat anti-mouse IgG/IgM antibody conjugated to alkaline phosphatase (AP) was used (Sigma-Aldrich, Italy, Cat. No. SAB3700987). Bound antibodies were

detected by adding the phosphatase substrate para-nitrophenyl phosphate (pNPP, Sigma-Aldrich, Italy) to the wells. Absorbance was measured at 405 nm using the Tecan M200 Infinite® Pro Microplate Reader (Tecan Life Sciences, Switzerland). ~~During data elaboration the negative control was subtracted from the value of absorbance.~~

1.4 Immunofluorescence

Cells were seeded at a density of 25000 cells over Deckglaser cover glasses 18 mm (Marienfeld, Germany) and cultured o/n. The day after, cells were fixed with paraformaldehyde solution 4% in phosphate buffer solution (PBS-PFA) (Sigma-Aldrich, Italy) and washed with 0.1 M of glycine (Sigma-Aldrich, Italy) and then with PBS without calcium and magnesium (Euroclone S.p.A., Italy). After fixation, non-specific binding sites were blocked with PBS Bovine Serum Albumine (BSA) 5% (Sigma-Aldrich, Italy) for 1 h at RT. After the blocking, cells were incubated with primary antibodies diluted in PBS BSA 1% for 1 h at RT. Cells were washed twice with PBS and incubated with the secondary antibody for 1 h at RT. Cells were then washed in PBS and incubated for 5 minutes with DAPI (Sigma-Aldrich, Italy) and washed with PBS, with distilled water and mounted using the FluorSave™ mounting medium (Merck, Germany) to Superfrost® Microscope Slides (Thermo Scientific, Thermo Fisher Scientific, Italy). Images were acquired using the Nikon Eclipse Ti-U microscope (Nikon Europe B.V.).

Organs and BXPC3 tumors from xenograft murine models were embedded into Killik O.C.T. (OCT) medium (Bio-Optica S.p.A., Italy) and maintained at -80 °C. A cryostat (Thermo Electron Corporation, MA, USA) was utilized to cut organs and tumors into slices of 7 µm, slices were subsequently mounted into SuperFrost® Plus (Fisher Scientific, Thermo Fisher Scientific, Italy) glass slides and stored at -80 °C. For immunofluorescence (IF), samples were fixed for 10 minutes with cold PBS-PFA, washed with PBS and blocked using a solution of 2% BSA, 0.25% casein from bovine milk (Sigma-Aldrich, Italy), and 0.1% gelatine from cold water fish skin (Sigma-Aldrich, Italy) in PBS for 1 h at RT. Slices were then incubated with primary antibodies diluted into blocking solution for 1 h at RT, then washed with blocking solution and incubated with secondary antibodies in blocking solution for 1 h at RT. Samples were further washed in PBS and incubated with DAPI 5 min at RT. Samples were washed twice with PBS and then with distilled water. Samples were mounted using FluorSave™ on high precision microscope cover glasses (Marienfeld, Germany). Images were acquired using the Nikon Eclipse Ti-U microscope.

1.5 Flow cytometry

For flow cytometry analysis, 300000 cells were blocked using PBS plus 2% BSA and then incubated with primary antibody diluted in the same blocking solution for 1 hour at RT under shaking. Cells were then washed twice with PBS plus 2% BSA and incubated with secondary antibody diluted in the same blocking solution for 1 hour at RT. The samples were washed and then resuspended into

PBS plus 2% BSA and 1% PFA. Samples were acquired using BD FACSCanto™II Cell Analyzer (Becton, Dickinson and Company, NJ, USA). Primary antibodies employed were: anti-GPC1 (Thermo Fisher Scientific, Italy, Cat. No. PA5-28055); AT101 25 μg/ml. Secondary antibodies employed were: anti-mouse IgM 488 conjugated (Bethyl, Fortis Life Science, MA, USA, Cat. No. A90-201D2) diluted 1:250; anti-rabbit IgG 488 conjugated (Bethyl, Fortis Life Science, MA, USA, Cat. No. A120-212D2) diluted 1:100. Data were analyzed using BD FACSDiva Software.

1.6 Labeling with cyanine 5.5

For the *in-vivo* biodistribution study, AT101 was conjugated with cyanine 5.5 (Cy5.5) (Cytiva, United Kingdom, Cat. No. PA25501), a dye that produces an intense signal in the near infrared region of the spectrum. One mg of AT101 was added to the Cy5.5 vial and the reaction was incubated for 30 minutes at RT, with additional mixing every 10 minutes. The labelled AT101 was separated from the excess free Cy5.5 by dialysis in PBS using a cellulose dialysis membrane with a cutoff of 14000 Da (Merck, Germany). Quantification of Cy5.5 was performed using a spectrophotometer (Cary 100 UV-VIS, Agilent) according to the guidelines in the protocol "Amersham fluorolink Cy5.5 monofunctional dye PA25501".

1.7 Hematoxylin Eosin staining

The tumor masses were collected and embedded in OCT. The frozen organs were then cut with the cryostat as described in the section "Immunofluorescence". Tumor slices were fixed in cold PFA-PBS for 10 minutes, washed in distilled water for 1 minute and stained with hematoxylin solution (Merck, Germany) for 3 minutes. Before eosin staining (Bio-Optica, Italy) for 45 seconds, the slices were washed in distilled water for 1 minute and incubated with 95% ethanol for 1 minute. Subsequently, the slices were dehydrated by serial incubation with ethanol 95% for 1 minute and by 2 incubations with ethanol 100%. Finally, the slices were incubated with xylene for 2 minutes and the cover glasses were mounted with Entellan™ (Merck, Germany). For IF, the sections were treated as described in the section "Immunofluorescence".

1.8 Statistical analyses

Statistical analyses were performed in RStudio (v4.0.5). The unpaired Student's t-test was used to calculate statistical difference between the means of two data sets. Survival function estimates between treatment groups were calculated with the Kaplan-Meier estimator using the package "survival"(v3.2-10); survival curves were plotted by Kaplan-Meier curves using the package "survminer"(v0.4.9). The log-rank test was used to calculate the p-values of the survival difference between the treatment groups using the package "survival" (v3.2-10). The comparison between the two groups (tumor growth curves) was performed using the 1-way analysis of variance test. $P < 0.05$ was considered statistically significant.

2 Additional Tables

Table 1. Primers employed for sequencing of VL gene

**Mouse VL specific primers 5’ to 3’**	
1	AGCAAGCGGCGCGCATGCCGAYATCCAGCTGACTCAGC
2	AGCAAGCGGCGCGCATGCCGAYATTGTTCTCWCCCAGTC
3	AGCAAGCGGCGCGCATGCCGAYATTKTGMTVACTCAGTC
4	AGCAAGCGGCGCGCATGCCGAYATTGTGYTRACACAGTC
5	AGCAAGCGGCGCGCATGCCGAYATTGTRATGACMCAGTC
6	AGCAAGCGGCGCGCATGCCGAYATTMAGATRAMCCAGTC
7	AGCAAGCGGCGCGCATGCCGAYATTCAGATGAYDCAGTC
8	AGCAAGCGGCGCGCATGCCGAYATYCAGATGACACAGA
9	AGCAAGCGGCGCGCATGCCGAYATTGTTCTCAWCCAGTC
10	AGCAAGCGGCGCGCATGCCGAYATTGWGTCSACCCAATC
11	AGCAAGCGGCGCGCATGCCGAYATTSTRATGACCCARTC
12	AGCAAGCGGCGCGCATGCCGAYRTTKTGATGACCCARAC
13	AGCAAGCGGCGCGCATGCCGAYATTGTGATGACBCAGKC
14	AGCAAGCGGCGCGCATGCCGAYATTGTGATAACYCAGGA
15	AGCAAGCGGCGCGCATGCCGAYATTGTGATGACCCAGWT
16	AGCAAGCGGCGCGCATGCCGAYATTGTGATGACACAACC
17	AGCAAGCGGCGCGCATGCCGACATCGAGCTCACCCAGTCTC
18	AGCAAGCGGCGCGCATGCCGACAGGCTGTTGTGACTCAGGAATC
**Mouse K primer 5’ to 3’**	
1	CAGTTGGTGCAGCATCAGCCCG

Table 2. Primers employed for sequencing of VH gene

**Mouse VH specific primers 5' to 3'**	
1	TTATCCTCGAGCGGTACCGAKGTRMAGCTTCAGGAGTC
2	TTATCCTCGAGCGGTACCGAGGTBCAGCTBCAGCAGTC
3	TTATCCTCGAGCGGTACCCAGGTGCAGCTGAAGSASTC
4	TTATCCTCGAGCGGTACCGAGGTCCARCTGCAACARTC
5	TTATCCTCGAGCGGTACCCAGGTYCAGCTBCAGCARTC
6	TTATCCTCGAGCGGTACCCAGGTYCARCTGCAGCAGTC
7	TTATCCTCGAGCGGTACCGAGGTGAASSTGGTGGAAT
8	TTATCCTCGAGCGGTACCGAVGTGAWGYTGGYGGAGTC
9	TTATCCTCGAGCGGTACCGAGGTGCAGSKGGTGGAGTC
10	TTATCCTCGAGCGGTACCGAKGTGCAMCTGGTGGAGTC
11	TTATCCTCGAGCGGTACCGAGGTGAAGCTGATGGARTC
12	TTATCCTCGAGCGGTACCGAGGTGCARCTTGTTGAGTC
13	TTATCCTCGAGCGGTACCGARGTRAAGCTTCTCGACTC
14	TTATCCTCGAGCGGTACCGAAGTGAARSTTGAGGAGTC
15	TTATCCTCGAGCGGTACCCAGGTTACTCTRAAAGWGTSTG
16	TTATCCTCGAGCGGTACCCAGGTCCAACTVCAGCARCC
17	TTATCCTCGAGCGGTACCGATGTGAACTTGGAAGTGTC
18	TTATCCTCGAGCGGTACCGAGGTGAAGGTCATCGAGTC
19	TTATCCTCGAGCGGTACCSAGGTSMARCTGCAGSAGTCWGG
**Mouse H primer 5' to 3'**	
1	GATTGGTTTGCCGCTAGCGACATTTGGGAAGGACTGA

Table 3. Aminoacid sequences of VL and VH chains of AT101

Heavy chain variable region:

QVQLQQSDAELVKPGASVKISCKASGYTFTDHAIHWVKQKPEQGLEWIGYISPGNGDIKY
NEKFKGKATLTADKSSSTAYMQLNSLTSEDSAVYFCKRYAYWGQGTLVTVSA

Light chain variable region:

DVLMTQTPLSLPVSLGDQASISCRSSQSIVHSNGNTYLEWYLQKPGQSPKLLIYKVSNRF
SGVPDRFSGSGSGTDFTLKISRVEAEDLGVYYCFQGSHVPWTFGGGTKLEIKP

3 Additional figure legends

Figure A1. Immunofluorescence analysis to evaluate GPC1 expression in BXPC3 and Jurkat using AT101 and the commercial anti-GPC1 antibody as positive control. In red the signal related to GPC1 protein is reported and in blue the signal related to the nuclei is reported. Scale bar: 25 µm.

Figure A2. Hematoxylin eosin staining to evaluate the possible presence of tissue damage. Purple blue refers to the nuclei, pink refers to the cytoplasms and the extracellular matrix. Scale bar: 100 µm.

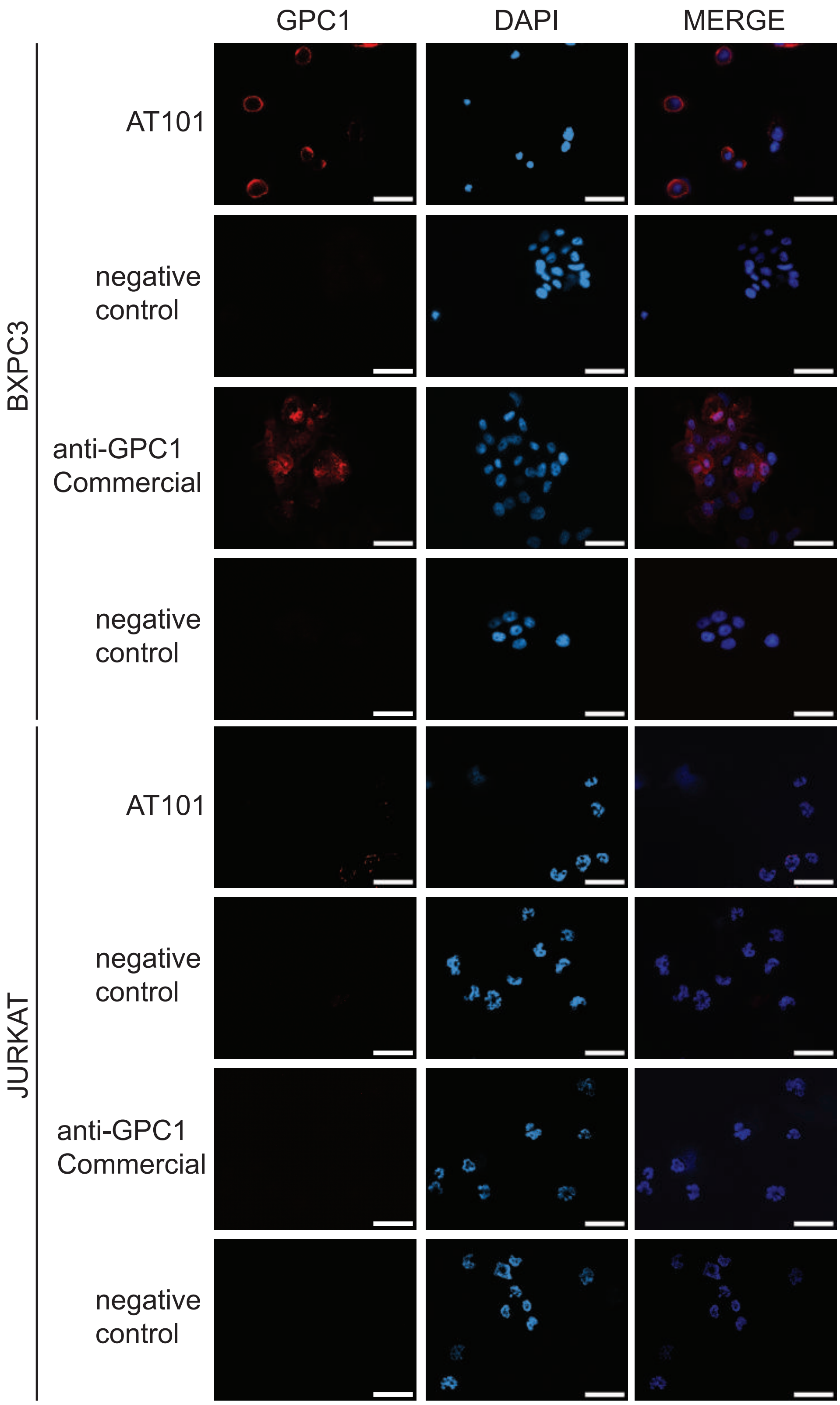
GPC1
DAPI
MERGE
BXPC3
AT101
negative control
anti-GPC1 Commercial
negative control
JURKAT
AT101
negative control
anti-GPC1 Commercial
negative control

FIGURE S1

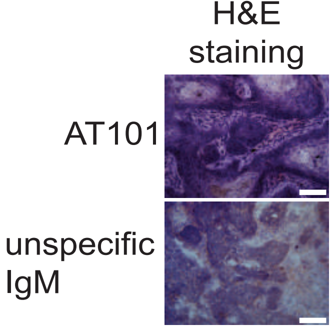

FIGURE S2
